# Supplementary material for: Mechanisms governing the pioneering and redistribution capabilities of the non-classical pioneer PU.1
Source: Nat Commun. 2020 Jan 21;11:402. doi: 10.1038/s41467-019-13960-2 (PMC6972792; doi:10.1038/s41467-019-13960-2)
Supplement: Supplementary file 7 — Source data [file 41467_2019_13960_MOESM7_ESM.zip › Source_Data/FigureS2/FigureS2B_MotifScanOutput/KmeansCluster7/homerResults/motif42.similar.html]

motif42

## Information for motif42

C
G
A
T
A
G
T
C
A
G
C
T
C
T
A
G
A
G
T
C
A
G
T
C
C
T
G
A
  
Reverse Opposite:  

A
G
C
T
A
C
T
G
A
C
T
G
A
G
T
C
C
T
G
A
A
C
T
G
G
C
T
A
  

|  |  |
| --- | --- |
| p-value: | 1e-30 |
| log p-value: | -7.032e+01 |
| Information Content per bp: | 1.881 |
| Number of Target Sequences with motif | 988.0 |
| Percentage of Target Sequences with motif | 30.78% |
| Number of Background Sequences with motif | 9461.4 |
| Percentage of Background Sequences with motif | 21.64% |
| Average Position of motif in Targets | 99.6 +/- 57.5bp |
| Average Position of motif in Background | 100.1 +/- 66.2bp |
| Strand Bias (log2 ratio + to - strand density) | 0.1 |
| Multiplicity (# of sites on avg that occur together) | 1.16 |
| Motif File: | file (matrix) reverse opposite |

### Similar de novo motifs found

|  |  |  |  |  |  |  |  |
| --- | --- | --- | --- | --- | --- | --- | --- |
| Rank | Match Score | Redundant Motif | P-value | log P-value | % of Targets | % of Background | Motif file |
| 1 | 0.796 | A G T C C G A T C T A G A G T C A G T C C T G A A C G T A C T G | 1e-21 | -49.454206 | 13.18% | 7.97% | motif file (matrix) |
| 2 | 0.798 | C T A G G A T C G A C T T C A G T C A G A G T C C G T A A C T G | 1e-16 | -37.206806 | 15.30% | 10.37% | motif file (matrix) |
